# Minor Structural Differences in the Cervical Spine Between Patients With Cervical Dystonia and Age-Matched Healthy Controls

**DOI:** 10.3389/fneur.2020.00472

**Published:** 2020-05-29

**Authors:** Petra Katschnig-Winter, Christian Enzinger, Dennis Bohlsen, Marton Magyar, Stephan Seiler, Edith Hofer, Sebastian Franthal, Nina Homayoon, Mariella Kögl, Karoline Wenzel, Hannes Deutschmann, Franz Fazekas, Reinhold Schmidt, Petra Schwingenschuh

**Affiliations:** ^1^Department of Neurology, Medical University of Graz, Graz, Austria; ^2^Division of Neuroradiology, Vascular and Interventional Radiology, Department of Radiology, Medical University of Graz, Graz, Austria; ^3^Institute for Medical Informatics, Statistics and Documentation, Medical University of Graz, Graz, Austria

**Keywords:** cervical dystonia, cervical spine, MRI, structural, inter-rater reliability

## Abstract

**Background:** Cervical dystonia is the most common form of focal dystonia. The frequency and pattern of degenerative changes of the cervical spine in patients with cervical dystonia and their relation to clinical symptoms remain unclear as no direct comparison to healthy controls has been performed yet. Here, we used magnetic resonance imaging (MRI) to investigate (1) whether structural abnormalities of the cervical spine are more common in patients with cervical dystonia compared to age-matched healthy controls, (2) if there are clinical predictors for abnormalities on MRI, and (3) to calculate the inter-rater reliability of the respective radiological scales.

**Methods:** Twenty-five consecutive patients with cervical dystonia and 20 age-matched healthy controls were included in the study. MRI scans of the cervical spine were analyzed separately by three experienced raters blinded to clinical information, applying different MRI rating scales. Structural abnormalities were compared between groups for upper, middle, and lower cervical spine segments. The associations between scores differentiating both groups and clinical parameters were assessed in dystonia patients. Additionally, inter-rater reliability of the MRI scales was calculated.

**Results:** Comparing structural abnormalities, we found minor differences in the middle cervical spine, indicated by a higher MRI total score in patients but no significant correlation between clinical parameters and MRI changes. Inter-rater reliability was satisfying for most of the MRI rating scales.

**Conclusion:** Our results do not provide evidence for a role of MRI of the cervical spine in the routine work-up of patients with cervical dystonia in the absence of specific clinical signs or symptoms.

## Introduction

Cervical dystonia (CD) is the most common form of focal dystonia, characterized by contractions of agonists and antagonists of the neck muscles, followed by twisting, repetitive movements, or abnormal posture (1). Cervical dystonia is very heterogeneous with regard to clinical presentation. This includes different forms of abnormal posture, isolated, or in combination. In addition, 28–68% of patients with cervical dystonia suffer from a head tremor (2–6) and 66–75% report neck pain of different intensity, which is responsible for a significant proportion of their disability (2, 7). Botulinum toxin injections are currently the first line treatment for cervical dystonia (8–10). However, the response can vary from excellent results to complete treatment failure.

Only few studies have systematically investigated the frequency of structural spinal changes in patients with CD by means of x-ray or computerized tomography (CT). Of 128 patients with CD who underwent cervical plain x-ray examinations, 63.1% showed degenerative changes (11). Another study found moderate to severe degenerative changes in CT scans of the cervical spine in 14 out of 34 patients with CD who were referred for selective peripheral denervation because of primary resistance to or secondary failure of botulinum toxin treatment (12). Previous studies using magnetic resonance imaging (MRI) have demonstrated that age-related changes of the cervical spine are widely present even in asymptomatic healthy subjects (13). Thus, the importance of degenerative changes in CD and how they relate to clinical symptoms remains somewhat unclear as no direct comparison to healthy controls has been performed.

The aim of this study was to use MRI to investigate (1) for the first time whether structural abnormalities of the cervical spine are more common in patients with CD compared to age-matched healthy controls, (2) whether an association exists between structural spinal changes and certain clinical parameters such as disease severity, and (3) to assess the inter-rater reliability of the respective radiological scales.

## Methods

### Participants

We investigated 25 consecutive patients (18 women, 7 men; mean age 60.9 ± standard deviation 12.1 years, range 28–75 years) with definite cervical dystonia either isolated (20 patients) or as part of a segmental dystonia (five patients) from the Movement Disorder outpatient clinic of the Department of Neurology, Medical University of Graz, Austria. With regard to the subtypes of cervical dystonia, the majority of our patients had an involvement of the neck and the head (80%), 20% had only neck involvement, none of our patients had a pure involvement of the head. Almost half of the patients had a torticollis/-caput (42.9%), followed by a combination of torticollis/-caput and laterocollis/-caput (28.6%), 19% of patients had a combination of torticollis/-caput and retrocollis/-caput, the minority had a pure laterocollis (4.8%) or a combined torticollis and anterocollis (4.8%).

We further recruited 20 age-matched healthy control subjects (14 women, 6 men; mean age 55.8 ± standard deviation 16.7 years, range 26–79 years) without clinical symptoms such as neck pain or brachialgia, and without a previous history of cervical spine disease or earlier interventions performed in the cervical region. Subjects with pseudo-dystonia, claustrophobia, or contraindications for MRI (pregnancy or metal implants) were excluded from this study. In the MRI scanner all patients were able to keep their head and neck in a neutral position so that blinding was respected. All patients receiving BTX treatment (24 patients) had their last injections at least 3 months before the clinical examination. Informed consent in accordance with the Declaration of Helsinki was obtained from all subjects and the study was approved by the ethics committee at the Medical University of Graz.

### Clinical Examination

In addition to demographics and taking the medical history, clinical examination included a standardized neurological examination focusing on signs and symptoms of root or spinal cord compression and the Tsui score (14) to evaluate the severity of cervical dystonia.

### MRI of the Cervical Spine

Study participants underwent MRI of the cervical spine at the Department of Radiology, Medical University of Graz, Austria at a single 3.0-T whole-body scanner (Trio; Siemens, Erlangen, Germany). The protocol included sagittal T2-weighted TSE (Turbo spin echo) sequences (TR 4,000, TE 112 ms, slice thickness 3 mm) and sagittal T1-weighted TSE sequences (TR 550, TE 11 ms, slice thickness 3 mm) in addition to axial sequences [T2-weighted TSE and 2D T2-weighted gradient-echo (me2d)]. No intravenous contrast agent was given. Nine patients had received a routine MRI of the cervical spine (1.5 Tesla) with a similar protocol within 3 months prior to the study visit at an external radiological clinic. After all scans had been collected three experienced raters (CE, DB, and MM), who were blinded to the diagnosis and other clinical or demographic information, independently analyzed the anonymized scans according to a standardized protocol. Raters evaluated each cervical spine segment (C2/C3 to C6/7) and the whole cervical spine (C2–C7) using six scales (Table 1), which focused on different radiologic abnormalities in sagittal images. In addition, axial sequences were checked for additional abnormal findings. According to the scale published by Kang et al. (15), T2-weighted sagittal images were used to assess cervical canal stenosis. Degenerative changes in the cervical intervertebral disk (disc degeneration by loss of signal intensity in T2, posterior, and anterior disc protrusion in T1, narrowing of the disc space and foraminal stenosis in T1) were evaluated by using Matsumotos' scale (16). Subscale posterior disc protrusion was omitted due to its similarity to Kang's scale. By means of the Modic score (17) MRI signal intensity changes in the vertebral body marrow adjacent to the endplates of degenerative disk in T1 and T2 were assessed. The primary outcome parameter was the MRI total score, a sum score of all six subscores for each segment to cover all aspects of MRI changes, secondary outcome parameters were the respective subscales.

**Table 1:** Radiological scales.

**Cervical Canal Stenosis [18]**	
0	Absence of central canal stenosis
1	Nearly complete obliteration of subarachnoid space, including obliteration of the arbitrary subarachnoid space exceeding 50%, without signs of cord deformity
2	Central canal stenosis with cord deformity but without spinal cord signal change
3	Presence of spinal cord signal change near the compressed level on T2-weighted images
**Degenerative changes in the cervical intervertebral disk [19]**	
Disc degeneration (Matsumoto 1^*****a*****^)	
0	Bright as or slightly less bright than cerebrospinal fluid
1	Dark and/or speckled
2	Almost black
Anterior disc protrusion (Matsumoto 2^*****a*****^)	
0	Disc material confined within the anterior margin of the vertebral body
1	Disc material protruding beyond the anterior margin of the vertebral body
Narrowing of the disc space (Matsumoto 3^*****a*****^)	
0	No narrowing or <25% loss in height compared with the most adjacent normal disc space
1	25–50% loss of height
2	More than 50% loss of height
Foraminal stenosis (Matsumoto 4^*****a*****^)	
0	No obliteration of intraforaminal fat
1	Disc material or bony spurs obliterating intraforaminal fat unilaterally or bilaterally
**Degenerative changes in vertebral bodies [20]**	
1	Hypointense signal in T1-weighted imaging and hyperintense signal in T2-weighted imaging corresponding to vertebral body edema and hypervascularity
2	Hyperintense signal in T1-weighted imaging and hyperintense signal in T2-weighted imaging reflecting fatty replacements of the red bone marrow
3	Hypointense signal in T1-weighted imaging and hypointense signal in T2-weighted imaging consisting of subchondral bone sclerosis

a * for simplification called Matsumoto 1–4*.

Based on clinical and functional relevance, structural abnormalities on MRI were compared between both groups for upper (C2/C3), middle (C3/C4 + C4/C5) and lower (C5/C6 + C6/C7) cervical spine using *t*-tests if data were normally distributed or Mann–Whitney *U*-tests in case of non-normal distribution. For this purpose, ratings of the three independent raters for each segment were averaged and summarized for middle and lower cervical spine. Results of the secondary outcome parameters were corrected for multiple comparisons using the False Discovery Rate (FDR) (18).

The associations between those scores that allowed differentiation between both groups and four clinical parameters [disease duration, TSUI total, TSUI subscale pain, BTX efficacy (maximum decrease of symptoms from 0 to 100%)] were assessed in patients with cervical dystonia using age and sex adjusted linear regression. *P*-values obtained from regression analyses were also adjusted for multiple comparisons using FDR. A *p*-value of < 0.05 was considered significant.

Inter-rater reliability (IRR) for MRI images of the cervical spine was calculated with the intraclass correlation coefficient (ICC) for cervical canal stenosis, disc degeneration, narrowing of the disc space, the Modic classification, the sum score of all subscales of Matsumoto (Matsumoto total score), and the MRI total score. Fleiss kappa (κ) value was used to evaluate inter-rater reliability of anterior disc protrusion and foraminal stenosis because of their binary nature.

Statistical analysis was performed using program SPSS (IBM Statistics for Windows, version 23; Armonk, NY, USA) and R [R Core Team (2015). R: A language and environment for statistical computing. R Foundation for Statistical Computing, Vienna, Austria. URL https://www.R-project.org/.]

All values are reported as mean ± standard deviation (SD) unless otherwise given.

## Results

### Demographics and Clinical Details

Data are provided in Table 2.

**Table 2:** Demographics and clinical data.

	**Patients (*n* = 25)**	**Controls (*n* = 20)**	***p*-value**
	**mean ± SD or number (%)**	**mean ± SD or number (%)**	
Female sex	18 (72%)	14 (70%)	*p* = 0.886
Age, y	60.9 ± 12.1	55.8 ± 16.7	*p* = 0.234
Age at onset, y	43.7 ± 16.1		
Disease duration, y	16.8 ± 10.9		
Isolated CD	20 (80%)		
Positive FH	9 (36%)		
Precipitating trauma	5 (20%)		
Tremor	18 (72%)		
BTX	24 (96%)		
Duration of BTX, y	7.4 ± 6.1		
BTX dosage, mu	434.8 ± 187.3		
BTX efficacy, %	56.3 ± 28.9		
Tsui total score	6.7 ± 3.0		
Tsui pain score	1.2 ± 1.2		

*y, years, CD, cervical dystonia; FH, family history; BTX, botulinum toxin; mu, mouse units; SD, standard deviation*.

### MRI Abnormalities and Correlation With Clinical Parameters

Comparing the MRI total score of the upper, middle, and lower cervical spine between patients with CD and healthy controls we only found significant differences in the middle cervical spine (C3/C4 + C4/C5) (Table 3). Based on this sum score covering all aspects of radiological changes, structural abnormalities in the middle cervical spine were more prominent in CD patients compared to controls.

**Table 3:** MRI total score for upper, middle, and lower cervical spine.

	**Patients (mean + SD)**	**Controls (mean + SD)**	***p*-value**
Upper cervical spine	2.88 ± 0.89	2.52 ±1.30	0.293
Middle cervical spine	8.90 ± 3.85	6.23 ± 4.64	0.039^*^
Lower cervical spine	10.54 ± 4.85	9.57 ± 6.26	0.560

SD, standard deviation,

* *p < 0.05*.

Further analysis of the respective subscales revealed differences in the middle cervical spine between both groups in Matsumoto 2 and Matsumoto total pointing to more severe degenerative changes in the intervertebral disk in CD patients. However, after correction for multiple testing, no significant result for these secondary outcome parameters remained (Table 4). No further abnormalities were detected by analysis of axial sequences.

**Table 4:** Kang, Matsumoto, and Modic scores for upper, middle, and lower cervical spine.

	**Patients (median [IQR])**	**Controls (median [IQR])**	***p*-value (uncorrected)**
**Kang**			
Upper cervical spine	0.00 [0.00–0.17]	0.00 [0.00–0.00]	0.376
Middle cervical spine	1.33 [0.67–2.33]	1.00 [0.08–1.58]	0.086
Lower cervical spine	2.00 [0.67–2.50]	1.33 [0.75–2.25]	0.73
**Matsumoto 1**			
Upper cervical spine	1.67 [1.17–2.00]	1.50 [0.75–1.91]	0.249
Middle cervical spine	3.00 [2.33–3.50]	2.17 [1.33–3.33]	0.123
Lower cervical spine	3.00 [2.00–3.33]	2.67 [1.08–3.58]	0.382
**Matsumoto 2**			
Upper cervical spine	0.00 [0.00–0.00]	0.00 [0.00–0.00]	0.692
Middle cervical spine	0.67 [0.33–1.33]	0.33 [0.00–0.67]	0.013^*^
Lower cervical spine	1.33 [0.67–2.00]	1.00 [0.67–1.67]	0.33
**Matsumoto 3**			
Upper cervical spine	0.00 [0.00–0.50]	0.00 [0.00–0.33]	0.247
Middle cervical spine	1.33 [0.50–2.33]	0.33 [0.33–1.33]	0.068
Lower cervical spine	2.00 [1.17–3.50]	1.33 [0.17–3.58]	0.568
**Matsumoto 4**			
Upper cervical spine	0.00 [0.00–0.00]	0.00 [0.00–0.00]	0.113
Middle cervical spine	0.50 [0.00–1.17]	0.17 [0.00–0.63]	0.174
Lower cervical spine	0.33 [0.00–1.00]	0.83 [0.00–1.00]	0.522
**Matsumoto total**			
Upper cervical spine	2.00 [1.67–2.00]	1.50 [0.75–2.00]	0.145
Middle cervical spine	5.67 [3.67–7.17]	3.67 [2.08–5.33]	0.025^*^
Lower cervical spine	6.67 [4.33–9.00]	6.83 [1.92–9.17]	0.529
**Modic**			
Upper cervical spine	0.00 [0.00–0.67]	0.67 [0.00–0.67]	0.616
Middle cervical spine	0.00 [0.00–1.67]	0.00 [0.00–0.25]	0.168
Lower cervical spine	1.00 [0.33–2.00]	1.00 [0.00–2.92]	0.991

IQR, interquartile range.

* *Results did not stand correction for multiple comparisons*.

Correlation of the MRI total score, Matsumoto 2, and Matsumoto total score of the middle cervical spine with clinical parameters only showed a positive result for the Matsumoto total score and the TSUI total score (*p* = 0.024). More prominent degenerative changes of the intervertebral disk were associated with more severe clinical presentation. After correcting for multiple comparisons however, no significant correlation remained (Table 5). No other correlations between structural MRI changes and clinical parameters were found.

**Table 5:** Correlations between radiological scores and clinical parameters.

		***p*-value (uncorrected)**
MRI total score	DD	0.151
	Tsui total	0.198
	Tsui pain	0.509
	BTX efficacy	0.192
Matsumoto 2	DD	0.911
	Tsui total	0.074
	Tsui pain	0.379
	BTX efficacy	0.795
Matsumoto total	DD	0.434
	Tsui total	0.024^*^
	Tsui pain	0.267
	BTX efficacy	0.661

DD, disease duration; BTX, botulinum toxin.

* *Result did not stand correction for multiple comparisons*.

### Inter-Rater Reliability of MRI Scales

The MRI total score showed excellent or almost perfect agreement (ICC ≥ 0.706) except for segment C2/C3 (ICC = 0.294). Inter-rater reliability for cervical canal stenosis (Kang) showed excellent agreement in segments C3/C4, C4/C5, C6/C7, and C2–C7 (ICC ≥ 0.624). All other segments showed maximally moderate agreement (ICC ≤ 0.533). With segment C2/C3 (ICC = 0.374) being the only exception, the Matsumoto total score showed excellent or almost perfect agreement (ICC ≥ 0.667). Excellent inter-rater reliability for disc degeneration (Matsumoto 1) and narrowing of the disc space (Matsumoto 3) was found in segments C3/C4, C5/C6, C6/C7, and C2–C7 (ICC ≥ 0.618). In all other segments moderate agreement was found (ICC ≤ 0.605). Segment C6/C7 for anterior disc protrusion (Matsumoto 2) (κ = 0.636) and segment C5/C6 for foraminal stenosis (Matsumoto 4) (κ = 0.506) showed substantial/moderate agreement. In all other segments maximally fair agreement was found (κ ≤ 0.395). IRR of the Modic classification revealed maximally moderate agreement in all segments (ICC ≤ 0.594).

## Discussion

In this study, we investigated structural MRI findings of the cervical spine in patients with cervical dystonia compared to age-matched healthy controls.

Comparing structural abnormalities, we only found minor differences between our patients and controls. Although the MRI total score of the middle cervical spine was significantly higher in patients than in controls indicating more severe structural abnormalities in this anatomical region (Table 3), analysis of the subscales did not reveal any significant differences after correcting for multiple comparisons (Table 4).

Data on structural imaging in cervical dystonia is rare. Chawda et al. (12) assessed the severity of degenerative changes from the clivus to the mid-cervical spine by CT in patients referred to selective peripheral denervation because of either primary or secondary failure to BTX treatment. Fourteen out of 34 patients (41.2%) had moderate to severe degenerative changes predominantly at C2/C3 and C3/C4 without any significant difference in clinical parameters such as age, gender, disease duration, disability, and pain compared to patients with either no or minimal changes. However, duration of inadequate treatment was longer, head mobility was more restricted, and head tremor was more severe in the latter group. Even though no control group was investigated, the changes were thought to be more than would be expected in an age-matched control group.

Frequent changes on x-rays were found by Risvoll and Kerty (11) in a study on the diagnostic value of imaging and laboratory investigations in typical cervical dystonia. Patients were investigated with cerebral CT/MRI, plain X-ray examination with functional x-rays, blood tests including antinuclear and anticardiolipin antibodies, ceruloplasmin, and cerebrospinal fluid. 63.1% showed degenerative changes and abnormalities included, e.g., spine curvature disorders, forward luxation, or degenerative changes. These changes were age-related and in concordance to our findings no clear and consistent relations between symptoms and changes seen on x-ray films were depicted. The authors concluded that there is little point in requesting expensive tests if no other neurological symptoms than typical cervical dystonia are present.

A comparison of structural MRI changes in patients with cervical dystonia and healthy persons who will presumably exhibit the process of natural aging is important for understanding the true long term impact of cervical dystonia on radiological findings in CD patients.

Structural changes in MRI imaging of the cervical spine are frequent in general and even more with increasing age. They probably develop due to degenerative changes on a physiological basis predominately at level C5/C6 or even lower without producing clinical symptoms; C2/C3 is least often affected. Higher cervical segments are associated with most rotation and little flexion/extension, as opposed to the lower cervical spine where flexion/extension predominates.

Kato et al. (19) found that the sagittal diameter of the spinal canal and both the cross-sectional area of the dural tube and the spinal cord tended to decrease with increasing age in more than 1,200 asymptomatic subjects.

A grading system for disc degeneration was first published by Matsumoto et al. (16). He investigated 497 asymptomatic subjects and found the frequency of all degenerative changes linearly increasing with age. Disc degeneration was most common (86% in women, 89% in men above age 60) and was associated with disc protrusion and narrowing of the disc space. Disc degeneration (herniation, annular fissure, nucleus degeneration) was also found to be very frequent (81, 85.9, and 95.4%, respectively) in 102 asymptomatic Korean subjects (20). In a recent study by Nakashima et al. (21) disc bulging increased with age in terms of severity, frequency, and number of affected levels. The incidence was already very high in subjects in their 20s (73.3% in males and 78% in females) and increased further from the 20s to the 50s.

The development of Modic changes (degenerative changes in the vertebral bodies) over time was prospectively studied by Matsumoto et al. (22). Modic changes increased from 4.5 to 13.9% at follow up after 11.6 years and were associated with age above 40 years, male gender, and pre-existing disc degeneration. Similarly, Mann et al. (23) found subjects with Modic changes 2.5 times more likely to develop disc herniations at the same level than subjects without Modic changes.

In comparison to the literature above, we found degenerative changes in patients more often at a higher level (middle cervical spine). This part of the cervical spine seems to be more vulnerable than lower segments in patients with cervical dystonia. In accordance with Chawda et al. (12), we assume that the continuous abnormal head movements in cervical dystonia seem to strain the upper/middle cervical articulations more than the lower cervical spine, as there is less mobility at lower levels.

Concerning clinical parameters, the only positive correlation between Matsumoto total score in the middle cervical spine and the Tsui total score as degree of dystonia severity did not stand correction for multiple testing. All remaining correlations did not reveal a significant result. Since literature on MRI of the cervical spine and cervical dystonia is scarce, there is hardly any data on the association of clinical symptoms with spinal MRI abnormalities in this group of patients. Chawda et al. (12) found, that the duration of inadequate treatment was longer, head mobility was more restricted, and head tremor was more severe in a group of patients with moderate or severe degenerative changes on CT compared to patients with no or minimal changes. In comparison to the patients included in the above mentioned study, our patients only had mild to moderate symptoms that were relieved at least to some extent by regular botulinum toxin injections.

In a prospective, 10-year follow up study in patients with whiplash injury compared to healthy controls no significant associations between the progression of degenerative MRI findings according to the Matsumoto scale and the clinical symptoms (neck pain, shoulder stiffness, headache, and arm pain/stiffness) were observed in either group (22). Since degenerative changes in our study were hardly different from healthy controls, this might explain why we did not find strong associations between structural abnormalities and clinical symptoms.

For most of the MRI rating scales and subscales (cervical canal stenosis, disc degeneration, narrowing of the disc space, Matsumoto total score, and MRI total score), inter-rater reliability was excellent or almost perfect in the lower segments of the cervical spine. In higher segments, the rating was more heterogeneous, most likely influenced by difficulties due to overlapping anatomical structures. Results for the Modic score, which classifies degenerative changes in the vertebral bodies next to spinal disk (17), were not as good. Originally and importantly, this classification was introduced for the lumbar spine, for which good inter-rater agreement has been described in various studies (24–30). So far only a few papers assessed inter-rater reliability of the Modic classification in the cervical spine (22, 23, 31). Apart from substantial agreement published by (31) (*K* = 0.73) Fleiss Kappa values were comparable to our results (*K* = 0.54 and 0.62, respectively). Except for individual lower segments, inter-rater reliability for the subscales anterior disc protrusion, and foraminal stenosis was only fair. Difficulties in rating foraminal stenosis may be due to the fact that we used sagittal images whereas axial images were used by Matsumoto et al. (22).

With this study, we did not aim to discriminate whether potential morphological changes may be the cause or the consequence of CD. The novelty of our study lies first in the usage of MRI to study structural changes of the cervical spine in CD patients and second in the comparison to asymptomatic controls. However, there are some limitations to this study. Since patients were enrolled consecutively, a minority of patients (20%) had their CD as part of a segmental dystonia. There is a substantial clinical overlap between cervical and segmental dystonia with neck involvement. A recent study in patients with cervical dystonia, either pure focal or as part of a segmental dystonia has shown, that the majority (77.2%) of patients with focal neck onset (78.5%) remained focal indeed, whereas 22.8% later spread to a contiguous body part defining segmental dystonia. Segmental onset with neck involvement (8.2%) or focal onset elsewhere with segmental spread to the neck (13.3%) was also not uncommon (32).

A larger sample size might have raised the level of significance of the structural differences observed in the middle cervical spine.

Due to the limited sample size we were not able to include all possible CD subtypes in our study cohort. Therefore, we cannot exclude, that specific CD subtypes and those with more severe CD or longer disease duration do actually have significant cervical spine changes on MRI assessment, compared to healthy controls. Also, all but one of our patients received botulinum toxin on a regular basis, therefore we cannot make a statement about the natural course of cervical dystonia and its impact on structural MRI abnormalities. To answer this questions larger studies including patients with all subtypes of CD and longer disease duration in a longitudinal study protocol over a longer period, ideally a few decades, would be desirable. Another limitation is that the healthy controls were slightly younger than patients, however this difference was not statistically significant.

With regard to the use of scanners with different field strengths it is important to emphasize, that the vast majority of our participants underwent a 3.0-T MRI, and only few of them had been scanned at lower field strengths. To the best of our knowledge, there is no convincing evidence in the literature that this might have affected the results of our study considering the radiological ratings done (15, 16).

According to the EFNS guidelines from 2011 (8) structural brain imaging is not routinely required when there is a confident diagnosis of a formerly called “primary dystonia” [genetic or idiopathic case in which dystonia is isolated and there is no consistent pathologic change (33)]. Our results do not provide evidence for a role of MRI of the cervical spine in the routine work-up of patients with CD in the absence of commonly accepted clinical signs or symptoms, since degenerative changes are generally not more severe than in healthy controls. Based on our finding that structural changes in cervical dystonia are commonly found in higher cervical segments compared to healthy controls, it is important to consider abnormalities like radiculopathy or myelopathy even in atypical neurological distributions.

## Data Availability Statement

The datasets generated for this study are available on request to the corresponding author.

## Ethics Statement

The studies involving human participants were reviewed and approved by Ethics committee Medical University of Graz Graz, Austria. The patients/participants provided their written informed consent to participate in this study.

## Author Contributions

PS and CE contributed to conception and design of the study. PS, CE, and HD organized the study. CE, DB, and MM analyzed the scans. PK-W, SS, MK, KW, and PS contributed to the acquisition of clinical data. EH and PK-W were involved in design and execution of the statistical analysis. PK-W wrote the first draft of the manuscript, all authors revised the manuscript critically for important intellectual content and approved the submitted version.

## Conflict of Interest

The authors declare that the research was conducted in the absence of any commercial or financial relationships that could be construed as a potential conflict of interest.
